# Intracellular quantitative detection of human thymidylate synthase engagement with an unconventional inhibitor using tetracysteine-diarsenical-probe technology

**DOI:** 10.1038/srep27198

**Published:** 2016-06-02

**Authors:** Glauco Ponterini, Andrea Martello, Giorgia Pavesi, Angela Lauriola, Rosaria Luciani, Matteo Santucci, Michela Pelà, Gaia Gozzi, Salvatore Pacifico, Remo Guerrini, Gaetano Marverti, Maria Paola Costi, Domenico D’Arca

**Affiliations:** 1 University of Modena and Reggio Emilia, Department of Life Sciences, Via Giuseppe Campi 183, Modena, 41125, Italy; 2 University of Edinburgh, University/British Heart Foundation Centre for Cardiovascular Science, The Queen’s Medical Research Institute, Edinburgh, EH16 4TJ, UK; 3 University of Modena and Reggio Emilia, Department of Biomedical, Metabolic and Neural Sciences, Via Giuseppe Campi 287, Modena, 41125, Italy; 4 University of Ferrara, Department of Chemical and Pharmaceutical Sciences, Via Fossato di Mortara 17-19, Ferrara, 44100, Italy

## Abstract

Demonstrating a candidate drug’s interaction with its target protein in live cells is of pivotal relevance to the successful outcome of the drug discovery process. Although thymidylate synthase (hTS) is an important anticancer target protein, the efficacy of the few anti-hTS drugs currently used in clinical practice is limited by the development of resistance. Hence, there is an intense search for new, unconventional anti-hTS drugs; there are approximately 1600 ongoing clinical trials involving hTS-targeting drugs, both alone and in combination protocols. We recently discovered new, unconventional peptidic inhibitors of hTS that are active against cancer cells and do not result in the overexpression of hTS, which is a known molecular source of resistance. Here, we propose an adaptation of the recently proposed tetracysteine-arsenic-binding-motif technology to detect and quantitatively characterize the engagement of hTS with one such peptidic inhibitor in cell lysates. This new model can be developed into a test for high-throughput screening studies of intracellular target-protein/small-molecule binding.

In anticancer research, chemical and clinical observations have prompted the development of new translational medicine concepts with applications in the early phases of a large number of clinical trials. The anticancer drugs identified to date still show a high degree of clinical failure, often in the late stages of the trials^1^. Thus, more effective strategies have been actively pursued. Drug discovery has shifted from the conventional concept of cytotoxic chemotherapy to targeted therapy; that is, the development of agents that target molecules and signal transduction pathways that are aberrant in cancer cells^2^,^3^,^4^. In this context, proving the candidate drug’s interaction with its target protein in live cells, which is a prerequisite for the effectiveness of therapeutic drugs, is of central relevance to the successful outcome of the drug discovery pipeline^5^,^6^,^7^. Moreover, the availability of tools to measure drug-target engagement in a biological environment would provide useful information for improving the understanding of the physicochemical aspects of drug-target interactions. To this aim, we propose a method based on cells that ectopically express a human thymidylate synthase (hTS) enzyme engineered to sustain the FRET-based monitoring of hTS-inhibitor binding at the cell lysate level.

More than 1600 clinical trials are currently ongoing to explore different applications of folate-targeted enzymes in anticancer therapy. In many of these trials, hTS-targeted chemotherapeutics are at the forefront as single agents or in combinations^8^,^9^,^10^,^11^. In the dimeric form, the TS enzyme catalyses the reductive methylation of deoxyuridine monophosphate (dUMP) to dTMP and provides the sole de novo pathway to thymidine production for DNA synthesis^12^. Thus, its inhibition causes the depletion of cell growth by impairing DNA replication and repair. The failure of hTS monomers to regulate hTS mRNA with ensuing hTS overexpression is one the mechanisms of the onset of resistance to TS-targeted drugs^13^,^14^,^15^. Following the identification of hotspot residues at the intermonomer interface that are crucial for the stability of the dimeric assembly^16^, we discovered several octapeptidic inhibitors that, differently from classical hTS inhibitors, bind the protein dimer at the monomer/monomer interface and stabilize its inactive conformation^17^,^18^. Among these inhibitors, peptide LSCQLYQR (LR) was shown to accumulate in cells to steady-state concentrations of several tens of micromoles/litre^19^ and was able to inhibit hTS and cancer cell growth without causing the overexpression of the enzyme^17^,^20^.

According to a strategy devised by Tsien and co-workers, some fluorogenic biarsenicals can covalently bind proteins that contain a tetracysteine motif, CCXXCC, thereby strongly enhancing their emission quantum yield and making such tetracysteine-containing proteins observable within cells^21^,^22^. The CCPGCC sequence features a hairpin conformation that optimizes the covalent binding of each As atom of the probe to the S atoms of two cysteine units. Apparent dissociation constants as low as 4 pM have been measured for complexes formed between the fluorescein diarsenical probe FlAsH and some dodecapeptides that include this sequence^23^. This strategy has demonstrated great value in investigating protein-protein interactions and protein structural modifications^24^,^25^. However, its potential for detecting the intracellular binding of a target protein with a candidate drug has apparently been overlooked.

In the present work, we demonstrate this potential through a test case involving hTS and its peptidic inhibitor LR. We adapted the tetracysteine-arsenic-binding-motif technology to enable the quantitative characterization of the binding of hTS with the LR peptide in a cell lysate environment (see Fig. 1 for an overview). We modified hTS by introducing a CCPGCC hexapeptide (TC motif) that is able to covalently bind the green-emitting fluorescein-based diarsenical probe (FlAsH-EDT_2_)^21^,^22^, and the LR peptide was conjugated to a blue-emitting probe (hilyte405, h). Due to a favourable spectral overlap, hilyte405 can efficiently sensitize the FlAsH emission by non-radiative energy transfer (FRET) in the hTS-TC-FlAsH/LR-h complex, thus revealing hTS/LR engagement in a cellular environment.

## Results

### Transfected HEK293 cells express enzymatically active tetracysteine-hTS

The TS activity depends on the structural integrity of the C-terminal region, and the proteasomal degradation of human TS is ubiquitin-independent and mediated by an intrinsically disordered region at the N terminus of the molecule^26^. Therefore, to avoid interference with these processes, we inserted the CCPGCC arsenic-binding domain construct at the N terminus at position aa 20–21 from the predicted start. The engineered hTS was expected to be ectopically expressed and preserve its activity inside cells. Indeed, the small CCPGCC motif is less likely to interfere with hTS structure and biological functions than other commonly used tags (CFP, YFP, eGFP)^27^. An overview of the cloning strategy is given in the supplementary material.

**Figure 2:**
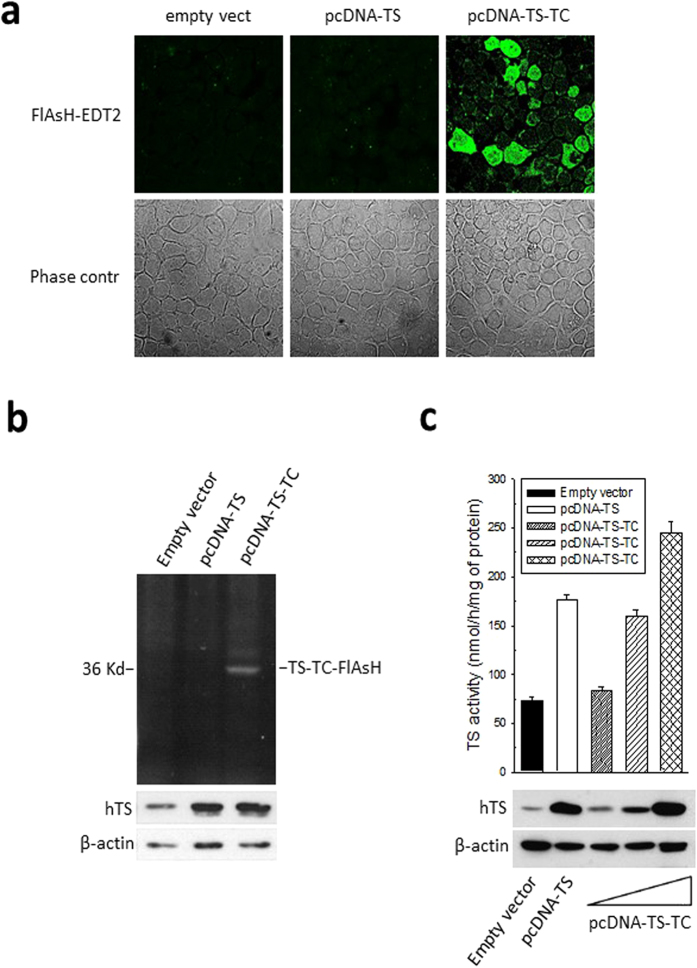
Characterization of the tetracysteine hTS tag. (**a**) Confocal fluorescence microscopy images of HEK293T cells transfected with empty vector, pcDNA-TS or pcDNA-tetracysteine-hTS (pcDNA-TC-TS) showing the green fluorescence of FlAsH bound to the recombinant TC-hTS protein; (**b**) A representative cropped polyacrylamide-gel for hTC-TC-FlAsH (the full-length gel is presented in Supplementary Fig. 2). The gel was run using different sample preparations: 10 mM TCEP (tris(2 carboxyethyl)phosphine) as the reductant instead of BME (β-mercaptoethanol) was added to the lysate before FlAsH-EDT2 labelling. The bottom panel shows the western blot for hTS protein expression using the same samples, but with BME used as the reductant; (**c**) evaluation of hTS activity in HEK293T cells ectopically expressing hTS and TC-hTS; top: TS activity, bottom: TS protein levels. Error bars represent s.d. (n = 3).

HEK293T cells were transiently transfected with either pcDNA3.1-empty vector, or pcDNA3.1-hTS or pcDNA3.1-tetracysteine-hTS. Following treatment with FlAsH-EDT_2_, we observed the expected green fluorescence enhancement only in TC-hTS-transfected cells (Fig. 2a). We confirmed the specific binding of FlAsH-EDT_2_ to the recombinant TC-hTS by loading the polyacrylamide gel with the proteins extracted from HEK293T cells transiently transfected with the three vectors described above and treated with FlAsH-EDT_2_. The fluorescence emission of the TC-bound FlAsH was captured, and the ectopic expression of hTS was evaluated by immunoblot analysis (Fig. 2b, Supplementary Fig. 2). The ectopically expressed TC-hTS protein preserved its enzymatic activity. As shown in Fig. 2c, the HEK293T cells transfected with pcDNA3.1 empty vector displayed basal hTS activity. The hTS ectopic expression (pcDNA3.1-hTS) increased enzyme activity by 2.3 times. A dose-dependent induction of activity was also observed in cells ectopically expressing increased amounts of engineered TC-hTS (pcDNA3.1-tetracysteine-hTS), thus demonstrating that the enzyme activity increased together with the quantity of internalized plasmid. Under the same conditions, hTS protein levels, as assessed by western blot analysis, increased in parallel with hTS activity.

### FRET experiments enable the quantitative characterization of LR-peptide binding to recombinant hTS

**Figure 3:**
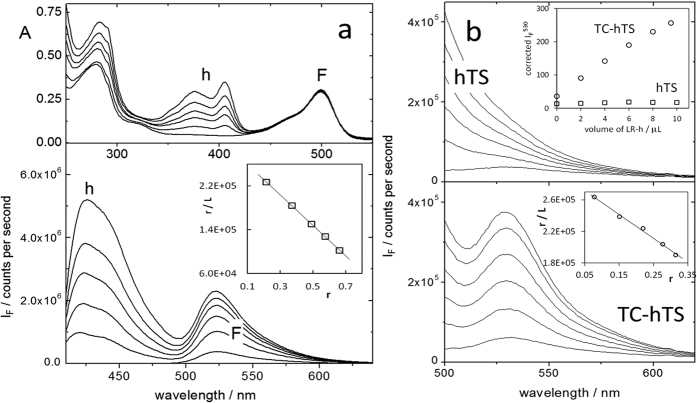
Fluorometric analysis of hTS/LR binding. (**a**) top: UV-visible absorption spectra of a 4.2 μM hTS solution tagged with fluorescein (F, 3.8 μM) with subsequent additions of LR-hilyte405 (h); bottom: emission spectra of hilyte405 (h) and fluorescein (F) upon excitation at 370 nm of solutions in phosphate buffer containing a fixed hTS-F concentration and increasing concentrations of LR-h. Inset: Scatchard plot for the hTS-F/LR-h fluorometric titration (r = fraction of occupied binding sites, L = concentration of free LR-h ligand). (**b**) FlAsH emission upon hilyte405 excitation at 380 nm for lysates of hTS-expressing cells (top, hTS) and hTS-tetracys-transfected cells (bottom, TC-hTS). Insets: corrected FlAsH emission intensity at 522 nm as a function of added LR-h volume and the corresponding Scatchard plot.

The fluorometric analysis of the LR/hTS interaction proceeded in three steps. We first investigated the binding of the peptide to the recombinant protein in buffer. Then, we compared these results with the behaviour displayed by the octapeptide versus hTS in two cellular environments of increasing complexity. For the first experiment, a recombinant hTS tagged with fluorescein (details are provided in ref. 28 and in the Methods section) was titrated with the LR-h conjugate. The tagged LR peptide was tested versus recombinant hTS and showed inhibition with an IC_50_ of 7 ± 1 μM (Supplementary Fig. 3), consistent with the results observed for inhibition by LR^17^. The UV-visible absorption results presented in Fig. 3a show the progressive addition of LR-h (maximum at 404 nm) to a solution of 4.2 μM hTS tagged with fluorescein (hTS-F, maximum at 495 nm, 3.8 μM) in phosphate buffer at pH 7.5. Along the course of titration, excitation of hilyte405 (h) caused emissions from h itself, with a maximum at 420 nm, and from fluorescein (F), with a maximum at 522 nm. The latter resulted from both direct excitation and energy transfer from h (Fig. 3a).

For a given LR-h concentration, the I_420_/I_522_ emission ratio was independent of the 4 × 10 mm^2^ cuvette orientation and, therefore, of the average path length of emitted light through the solution. This result indicates that radiative energy transfer, i.e., the reabsorption of h emission by F, is negligible and that the observed FRET derives from non-radiative energy transfer between the h donor and the F acceptor within LR-h/hTS-F complexes. The h emission increased linearly with the amount of LR-h added, as is expected if LR-h occurs in excess with respect to the complex. By contrast, the F emission showed a saturating trend, typical of titrations, and monitored LR-h/hTS-F complex formation. Scatchard analysis of the fluorescence data (inset in Fig. 3a) yielded a good linear fitting corresponding to a 1:1 binding (-intercept/slope = 1.03) and an equilibrium constant for the hTS-F/LR-h complex dissociation, K_d_ = 3.6 μM (±10%). We obtained the best fitting with a saturation value for the protein/peptide complex concentration of 3 μM, which was 29% lower than the total protein concentration. This result implies that only part of the protein can bind the peptide, a finding consistent with the calorimetric results reported in ref. 17 and interpreted by postulating that only the di-inactive form of the protein (approximately 25% of the total hTS protein) can bind the LR peptide (experimental work aiming at closely testing this assumption is currently underway).

### LR-h/hTS-TC-FlAsH FRET proves enzyme/inhibitor binding in cell lysates and enables quantitative characterization

In a step-by-step increase in matrix complexity, we performed similar experiments in HEK293T cell lysates to which hTS-F had been added up to a concentration of 2.5 μM. Again, the h emission increased linearly with the LR-h concentration, whereas the F emission intensity showed saturating behaviour (Supplementary Fig. 4). The difference here was that the latter emission showed a delayed initial increase, consistent with initial binding of a fraction of LR-h to a species different from hTS. Indeed, the Scatchard plot showed that the first two points deviated from the linear trend (Supplementary Fig. 4). However, if the equilibrium model was corrected to include a tight LR-peptide binder (K = 5 × 10^7 ^M^−1^) at a 150 nM concentration (see the Methods for details), a good linear plot was recovered with a K_d_ and a protein/peptide binding ratio indistinguishable from those obtained in phosphate buffer. Again, the saturation protein/peptide complex concentration was approximately 20% lower than the spectrophotometric protein concentration.

We finally characterized the LR/hTS binding directly in lysates of HEK293T cells ectopically expressing TC-hTS. We monitored the formation of the protein/peptide complex by observing FRET from hilyte405, bound to the LR peptide, to the diarsenical probe FlAsH added to the cell lysates to tag the hTS protein fused with the TC motif. We performed four subsequent additions of a concentrated FlAsH solution, each corresponding to a final concentration 0.15 μM, to the lysates of hTS- and of TC-hTS expressing HEK293T cells. The FlAsH emission (excitation at 480 nm) increased with the added amount of FlAsH and, in the case of the hTS-expressing cells, continued to increase for approximately forty minutes after the addition of the probe. Indeed, binding of the two arsenic atoms to the four cysteines of the motif may require several tens of minutes^29^. After the fourth addition, at a total FlAsH concentration of 0.6 μM, we obtained an emission from the TC-hTS cell lysate that was 2.5 times higher than that from the hTS cell lysate (Supplementary Fig. 5). We then performed five additions of LR-h to these solutions up to a final concentration of 2.6 μM. We observed FRET from the h donor to the FlAsH acceptor in TC-hTS lysates, but not in hTS lysates (Fig. 3b). After a straightforward correction of the FlAsH emission intensity for the h emission tail, we analysed the data according to the Scatchard method. We obtained a good linear plot (inset in Fig. 3b) using a saturation protein/peptide complex concentration 2.5 μM and a K_d_ of 3.4 μM, which was in excellent agreement with the K_d_ value obtained with the simplified model in phosphate buffer and with the inhibition data given above for the LR-h conjugate and published for the LR peptide^17^. In this case, the point corresponding to the first LR-h addition could be aligned with the following points by including a 30 nM tight LR binder, which was present in the TC-hTS cell lysate, to the overall equilibrium model.

In summary, we developed HEK293T-transformed cells that ectopically expressed an engineered hTS with preserved activity and stability. The TC motif fused in this protein can bind a diarsenical fluorophore, which is able to act as an electronic energy acceptor within complexes involving the engineered protein and a small peptidic inhibitor tagged with an energy donor, thus providing a signal that can be used to recognize and quantitatively characterize the protein/inhibitor interaction in cell lysates. Although TC-diarsenical probe technology has been applied in problems involving protein-protein interactions and protein structural modifications^24^, to our knowledge, this is its first application to the FRET-based monitoring and quantitative characterization of the interaction of a target protein with a small-molecule drug candidate in cell lysates. This recombinant model is easy to apply and offers an unprecedented opportunity to perform a displacement assay for the screening of specific inhibitors of the hTS monomer/monomer interaction in cells. Work in this direction is ongoing. Of more general interest, the approach described here can be adapted to develop assays to quantitatively screen druggable protein/small-molecule interactions in cells. Finally, simple sequential application to a cellular sample of two different diarsenical probes, e.g., green-emitting FlAsH-EDT_2_ and red-emitting ReAsH-EDT_2_, and the detection of their emissions over time will provide a simple means to follow the turnover and trafficking of hTS or any other suitably engineered protein.

## Methods

### Synthesis of LR-hilyte405

The conjugation of LR to hilyte405 was achieved using the classic thiol-Michael reaction. A solution of fluorescent probe (1 mg, 1 equiv.) in CH3CN (250 μL) was added to a stirred solution of the LR peptide (1.1 equiv.) in 250 μL of H_2_O, followed by the addition of 25 μL of NaHCO_3_ 5%. The mixture was stirred in the dark under a nitrogen atmosphere at ambient temperature for 15 minutes. The reaction was monitored by HPLC and MS analysis. After completion of the reaction, HPLC purification produced the fluorescent conjugate in quantitative yield.

### Protein Purification

The human *thymidylate synthase* (hTS) protein was obtained from a culture of *E. coli* (DH5α) carrying the plasmid vector pQE80L_hTS cloning protein-encoding gene into the EcoRI/BamHI restriction sites of plasmid vector pQE80L (*Addgene*)^16^. Positively transformed cells were selected by ampicillin resistance (100 μg/mL) and cultured by being shaken in 1 L of LB-growth medium at 37 °C and 120 RPM for 6 hrs. Isopropyl β-D-1-thiogalactopyranoside (*IPTG*, 1 mM) was added to the bacterial culture at 37 °C and 120 RPM for 14 hrs. After the induction of protein expression, the entire cell culture was centrifuged for 20 mins at 4 °C and 4500 RPM. The cellular pellet was resuspended with *buffer-A* (NaCl 30 mM, NaH_2_PO_4_ 20 mM, pH 7.0). Protease inhibitor (*Roche*) was added to the cellular suspension and bacterial cells were lysed by sonication (*Melsungen Labsonic*). The suspension was centrifuged at 12000 RPM, at 4 °C, for 20 mins and the lysate was filtered through 0.45/0.2 μm syringe filters. The filtered lysate sample was incubated with Nickel resin for 1 h on ice at 120 RPM. Two washing steps were performed: three volumes of buffer-A and then three volumes of *buffer-B* (NaCl 30 mM, NaH_2_PO_4_ 20 mM, imidazole 100 mM, pH 7.0). The target protein was eluted with *buffer-C* (NaCl 30 mM, NaH_2_PO_4_ 20 mM, Imidazole 1 M, pH 7.0) using a flow rate of 1 mL/min. A desalting step with a HiTRAP-Desalting column (5 mL, *GE Healthcare*) into the buffer-A was needed to remove imidazole. The enzyme’s kinetic activity was detected by the spectrophotometric method in each collected elution fraction. The purity level of the purified protein pool was checked by SDS-PAGE (≫95%). The enzyme’s kinetic profile was characterized (see below).

### Kinetic Characterization of the Enzyme

kcat and Km values were determined using a spectrophotometric method (Spectramax 190, Molecular Devices) based on the time-dependent increase in absorbance at 340 nm due to dihydrofolate (DHF) formation deriving from 5,10-methylenetetrahydrofolate (mTHF) enzyme-catalysed oxidation reaction. The reaction mixture contained 600 μL kinetic buffer (NaCl 30 mM, NaH_2_PO_4_ 20 mM, pH 7.0), 50% (v/v) of TES buffer (N-[tris(hydroxymethyl)methyl]-2-aminoethanesulphonic acid 100 mM, MgCl_2_ 50 mM, Formalin 13 mM, EDTA 2 mM, β-mercaptoethanol 150 mM, pH 7.4), hTS protein enzyme, mTHF and deoxyuridine-monophosphate (dUMP) substrates. The enzyme reaction was started when dUMP was added to the reaction mixture. Six enzyme concentration ranges (0.08–0.16–0.25–0.30–0.38–0.46 μM) and fixed mTHF and dUMP substrate concentrations of 55 μM and 120 μM, respectively were considered. The resulting kcat was equal to 0.9544 (s^−1^), in agreement with data reported in literature^17^. The mTHF Km value was determined considering a range of values of 3.50–6.90–8.65–20.70–41.50–69.20 μM and fixed enzyme and dUMP concentrations, namely 0.30 μM and 120 μM, respectively. The dUMP Km value was determined considering substrate concentrations of 5.15–10.29–20.58–41.16–80.61–161.21 μM and fixed enzyme and mTHF concentrations of 0.30 μM and 55 μM, respectively. The resulting Km values for mTHF and dUMP were equal to 5.87 μM and 9.74 μM, respectively, in agreement with data reported in the literature^17^. kcat and Km values were determined by analysing raw data with steady-state Michaelis-Menten kinetics. For each single determination assay, the experimental data were analysed in triplicate with a *p-value* < 0.005 representing statistical significance.

### Enzyme Inhibition Assay

The capability of LR-hilyte405 to inhibit the kinetic activity of hTS was evaluated. A spectrophotometric assay on a 96-well plate using a Spectramax-190 multiplate reader (*Molecular Device*) was performed. The assay consisted of monitoring, in the presence and absence of the inhibitor, the absorbance signal variation at DHF specific wavelength of 340 nm, for a total kinetic time of 180 sec at RT in a final volume of 100 μL per well. The inhibition reaction mixture included the following components: kinetic buffer (NaCl 30 mM, NaH_2_PO_4_ 20 mM, pH 7.0), 100 uL; hTS enzyme, at a concentration value to achieve an enzyme kinetic value in the 0.07–0.08 ΔOD/min range; the inhibitor compound, at several different concentration values; 50% (v/v) TES-Buffer, mTHF and dUMP substrates at concentrations corresponding to 10-times the Km value (saturating conditions). To measure the concentration of the inhibitors that reduce the activity of hTS by 50% (IC_50_), the assay was performed as follows. The ligand was dissolved in DMSO to obtain an initial concentration of 10 mM. A first inhibition assay at two single point concentrations was performed to identify the optimal ligand values to consider in the IC_50_ assay. LR-hilyte405 was tested at two concentrations (50 and 100 μM) in duplicate, with a statistically significant *p-value* < 0.005, to evaluate the inhibitory activity both at time zero and after an incubation of 1 h with hTS enzyme protein at RT and 50 RPM. The LR-hilyte405 peptide-probe conjugate was tested at 50 and 100 μM both at time zero and after 1 h incubation, which presented an inhibitory activity equal to 80% and 88%, respectively. Based on the obtained inhibition data, the ligand IC_50_ was measured using an enzyme concentration equal to 0.30 μM and substrate concentrations of 55 and 100 μM for mTHF and dUMP, respectively. Ten concentrations of LR-Hilyte 405 (0–2–5–10–15–25–50–75–100–125 μM) were tested in duplicate, with a statistically significant p-value < 0.005. The data were analysed in a Dixon-type plot format (v^−1^/[LR-h]) using a linear least-squares fitting (inset in Supplementary Fig. 3). The IC_50_, i.e., the LR-Hilyte 405 concentration at which the reaction rate was half the rate in the absence of inhibitor, and the associated error were obtained from the fitting parameters and their errors.

### cDNA Manipulations and Mutagenesis

hTS cDNA was amplified by PCR from reverse transcribed cDNA from a 2008 cervical cancer cell line using KAPA HiFi DNA polymerase (KAPABIOSYSTEMS) and the following primers. Primer1: 5′-GCGGAAGGGGTCCTG-3′ and Primer 2b Kpn1: 5′-CTCGGTACCGACGAATGCAGAACACTTCTTTATTATAGC-3′. The resulting 1.559-bp fragment consisting of the last 115-bp upstream the ATG codon, the entire cds and 3′UTR was cloned into the pTargeT™ Mammalian Expression Vector (Promega) generating the hTS -pTargeT plasmid. The CCPGCC amino acid sequence (tetracysteine motif) was introduced between the 20th and 21st amino acid via PCR-mediated cloning from the 2008 cDNA library. The fragment (a) from 115 bp upstream the ATG to 60 bp downstream was obtained using Primer1: 5′-GCGGAAGGGGTCCTG-3′ and primer 2a XmaI: 5′-ACAACAGCCCGGGCAACACCGCTCCTGTGCGGC-3′, containing the XmaI site inserted into the tetracysteine nucleotide sequence.

Primer1b XmaI: 5′-TGTTGCCCGGGCTGTTGTGACGCCGAGCCGCG-3′ including the same tetracysteine nucleotide sequence and primer2b KpnI:

5′-CTCGGTACCGACGAATGCAGAACACTTCTTTATTATAGC-3′ were used to generate a 1400-bp fragment (b) consisting of the remaining protein coding nucleotide sequence and the whole 3′UTR. Both fragments were cloned into the pTargeT™ Mammalian Expression Vector. Fragment (b) was subcloned into the XmaI and KpnI site of the vector-containing fragment (a) to generate the hTS-TC pTargeT construct. Then, hTS and hTS-TC were subcloned into the NheI and KpnI site of the pcDNA3.1(+) vector to generate the hTS-pcDNA3.1 and hTS-TC-pcDNA3.1 constructs.

### Cell culture and transfections

HEK293T cells were maintained in DMEM supplemented with 10% FCS, 1 mM glutamine, 100 U/mL penicillin, and 100 U/mL streptomycin (Life Technologies) in a humidified incubator with 5% CO_2_. For the expression of proteins, cells were grown on chamber slides and transfected with appropriate plasmid DNA using Lipofectamine™ LTX Transfection Reagent (Life Technologies) according to the manufacturer’s instructions.

### FlAsH Staining and Imaging

HEK293T cells (3 × 10^4^) were plated into 6-well microslides pre-coated with 0.01% (w/v) poly-L lysine and transfected the following day with 2.5 μg of DNA using Lipofectamine™ LTX Transfection Reagent. After 24 h, cells were labelled for 30 min with 12.5 μM 1,2-ethanedithiol (EDT) and 0.5 μM FlAsH (Life Technologies) in HBSS and then washed twice for 15 min in 150 μM 2,3-dimercaptopropanol (BAL) in HBSS. Cells were fixed with 4% (w/v) paraformaldehyde for 15 min at RT and then imaged using an inverted Leica TCS SP2 confocal microscope interfaced with an Ar Laser (488 nm/20 mW), equipped with AOBS and operated at a magnification of 63x (HCX PL APO 63x/1.40 - oil - Lambda blue correction). Images were processed using LCS Lite (Leica Microsystems, Heidelberg).

### SDS-PAGE of protein-FlAsH complexes

HEK293T cells (8.5 × 10^5^) were plated into 35-mm dishes and transfected the following day with 2.5 μg of empty vector, pcDNA-TS or pcDNA-TS-TC using Lipofectamine™ LTX Transfection Reagent. After 24 hours, the cells were harvested and lysed with RIPA buffer (15 μg total protein loaded per line). Laemmli sample buffer with SDS, glycerol, Tris-HCl pH 6.8, and bromophenol blue, but containing 10 mM TCEP as the reductant instead of BME or DTT, was added to the samples previously lysed by RIPA buffer. Samples were boiled at 100 °C for 5 min and cooled. Then, 10 μM FlAsH-EDT_2_ was added to the protein and sample buffer, incubated at room temperature for 15–30 min, loaded onto a 12% polyacrylamide gel, and run as usual. The protein-FlAsH complexes were visualized by placing the gel on a UV transilluminator equipped with a standard camera with the ethidium bromide filter selected on the camera.

### Western blotting

HEK293T cells were harvested from the substrate by scraping and pelleting before being lysed. Lysis was performed on ice for 15 minutes in RIPA buffer (1% sodium deoxycholate, 20 mM Tris-HCl (pH 7.6), 150 mM NaCl, 1% NP-40, 1 mM EGTA, 1 mM Na_2_EDTA, 1 mM Na_3_VO_4_, Na_2_MoO_4_, 1 mM PMSF and protease inhibitor cocktail (Sigma-Aldrich Corporation)). The supernatants were boiled with Laemmli buffer and β-mercaptoethanol at 95 °C for 5–10 minutes. Samples were then separated on a SDS-PAGE, followed by transfer to PVDF membranes. After transfer, membranes were blocked with 5% skim milk at room temperature for one hour. Membranes were stained with the indicated primary antibodies (the mouse monoclonal TS106 primary antibody (Abnova) and vinculin mouse antibody (Millipore, Inc.) in TBS-T with 5% dry milk overnight at 4 °C on a shaker. Horseradish peroxidase (HRP)-conjugated anti-mouse (sc-2005, Santa Cruz Biotechnology) secondary IgG antibodies were used, and the signal was visualized using the ECL Plus Western Blotting Detection System (GE Healthcare Biosciences).

### TS catalytic assay in cells

HEK293T cells (2.5 × 10^6^) were plated onto 100-mm dishes and transfected the following day with empty vector, pcDNA-TS (15 μg) or pcDNA-TS-TC (7, 13 or 20 μg) using Lipofectamine™ LTX Transfection Reagent. After 24 hours, the cells were harvested and split to analyse the protein levels of TS (using Western blotting) and to evaluate the TS activity. Cell pellets were thawed by the addition of ice-cold lysis buffer (200 mM Tris-HCl, pH 7.4, 20 mM 2-mercaptoethanol, 100 mM NaF and 1% Triton X-100), sonicated (three x 5 s), and subsequently centrifuged at 14,000 × g for 15 min at 4 °C. The supernatant was used for enzyme assays. The TS catalytic assay was performed according to a previously reported method. The assay determined the catalytic activity of TS by measuring the amounts of ^3^H released during the TS- catalysed conversion of [5-^3^H]dUMP to dTMP. Briefly, the assay was performed on the enzymes in assay buffer (lysis buffer without Triton X-100) and 650 μM 5,10-methylenetetrahydrofolate in a final volume of 50 μL The reaction was started by adding [5-^3^H]dUMP (1 μM final concentration, specific activity 5 mCi/mol), followed by incubation at 37 °C for 60 min, and stopped by adding 50 μl of ice-cold 35% trichloroacetic acid. Residual [5-^3^H]dUMP was removed by adding 250 μl of 10% neutral activated charcoal. The charcoal was removed by centrifugation at 14,000 × g for 15 min at 4 °C, and a 150-μl sample of the supernatant was assayed for tritium radioactivity by liquid scintillation counting in the liquid scintillation analyser Tri-Carb 2100 (Packard). For each cell line, the linearity of [5-^3^H]dUMP conversion with respect to the amount of protein and time was established.

### Lysate preparation for spectrofluorometric analysis

HEK293T cells (10 × 10^6^) were plated into 150-mm dishes (6 dishes were used for each condition) and transfected the following day with 25 μg of pcDNA-TS or pcDNA-TS-TC using Lipofectamine™ LTX Transfection Reagent; cells were collected 48 h post-transfection in 15-ml tubes and washed twice with 4 °C cold PBS for spectrofluorometric analysis. To avoid nuclear rupture and consequent chromatin release to obtain lysates as optically clear as possible, cellular pellets were re-suspended in 1.5 ml of hypotonic lysis buffer (20 mM Tris-HCl pH 7.5, 20 mM Na_2_HPO_4_, 30 mM NaCl, 1 mM TCEP, 10 mM HEPES pH 7.9, 0.01% NP-40, 1 mM PMSF, 1 mM Na_3_VO_4_, 1 mM Na_2_MoO_4_ protease inhibitor cocktail (Sigma-Aldrich Corporation)) and incubated for 45 min on a rotator at 4 °C to allow for the hypotonic swelling of cells and lysis by mechanical shearing. After centrifugation at 16,000 g for 30 min, supernatants for each sample ware collected and stored at −80 °C for further spectrophotometric analysis.

### *In vitro* FRET-based detection of hTS-LR interactions

UV-visible absorption measurements were performed with a Cary 100 Varian spectrophotometer. Fluorescence measurements were carried out with a Horiba Fluoromax-3 spectrofluorometer. Samples were contained in 4 × 10 mm^2^ quartz cuvettes. For experiments in phosphate buffer, hTS was tagged with fluorescein using the procedure described in ref. 27. 800 μl of a solution of fluorescein-tagged hTS (hTS-F) in phosphate buffer at pH 7.5 3.8 μM in fluorescein and 4.2 μM in hTS dimers were titrated with LR tagged with hilyte405 (LR-h), each addition corresponding to a final concentration between 0.15 and 0.2 μM. The fluorescein, hTS dimer and LR-h concentrations were determined spectrophotometrically using the following extinction coefficients: ε_max_ (fluorescein) = 79000 M^−1 ^cm^−1^, ε_280_ (hTS) = 89000 M^−1 ^cm^−1^, ε_280_ (fluorescein) = 30000 M^−1 ^cm^−1^,^28^ and ε_404_ (LR-h) = 35000 M^−1 ^cm^−1^, determined *ad hoc* for this work. The h (420 nm) and F (522 nm) emissions were measured by excitation at 370 or 380 nm. The F emission values were analysed according to the Scatchard method as functions of the LR-h concentration. The amount measured before LR-h addition and due to the direct excitation of F at 370 nm was subtracted from the intensity measured at 522 nm. However, because the addition of LR-h causes an increase in the absorbance at 370 nm, A370, the amount subtracted was reduced to take into account the effect of the LR-h inner filter by multiplying the value at [LR-h] = 0 by 10^−A370/2^. The contribution at 522 nm of the tail of the h emission was also subtracted. Thus, for each LR-h addition, the corrected intensity at 522 nm, I_522_, was only due to FRET and was therefore proportional to the concentration of hTS-F/LR-h complexes. It was used to calculate the fraction of occupied hTS binding sites, r, and the concentration of the free peptide ligand L using the extrapolated value at saturation, I_522_^∞^, as r = I_522_/ I_522_^∞^, L = L_t_-r[hTS]_t_, where [hTS]_t_ is the total concentration of protein that can bind the peptide. The value of this parameter that yielded the best fitting was usually somewhat smaller than the spectrophotometric total protein concentration. For the TS/LR complex, the Kd value was obtained as K_d_ = −1/s, where s is the slope of the linear least-square fitting of the Scatchard plot, r/L vs. r, and the protein/peptide binding ratio was -i/s, where i is the intercept and s the slope. Similar experiments were performed in 800 μl of the lysate of 60 × 10^6^ HEK cells to which hTS-F was added to a concentration of 2.5 μM (determined spectrophotometrically). To obtain a linear Scatchard plot, we complicated the binding model by adding an equilibrium describing the complexation of LR with a high-affinity binder present in the lysate. The goal was to prevent some of the peptide from binding to hTS, yielding a delayed increase in the I_522_ signal due to FRET within the hTS-F/LR-h complexes. Finally, LR-h was used to titrate the hTS protein present in the lysates of 60 × 10^6^ wild-type HEK cells and 60 × 10^6^ HEK cells transfected to express tetracysteine-modified hTS. Both lysates, 800 μl each, were first added with the diarsenical probe FlAsH. Four 2-μl additions were made to a final concentration of 0.6 μM. FlAsH emission was measured over time after each addition to allow the probe to bind to the tetracys motifs. Then, the lysates were titrated with LR-h and the h-to-FlAsH FRET was monitored upon h excitation at 370 nm. The resulting intensities at 530 nm, corrected as previously described for the fluorescein emission at 522 nm, were used in a Scatchard analysis of the two datasets parallel to that described above. In all cases, the errors associated with the K_d_ values were obtained from the errors associated with the slopes of the plots in the linear least-squares analyses and standard error propagation considerations.

## Additional Information

**How to cite this article**: Ponterini, G. *et al.* Intracellular quantitative detection of human thymidylate synthase engagement with an unconventional inhibitor using tetracysteine-diarsenical-probe technology. *Sci. Rep.* **6**, 27198; doi: 10.1038/srep27198 (2016).

## Supplementary Material

Supplementary Information

## Figures and Tables

**Figure 1:**
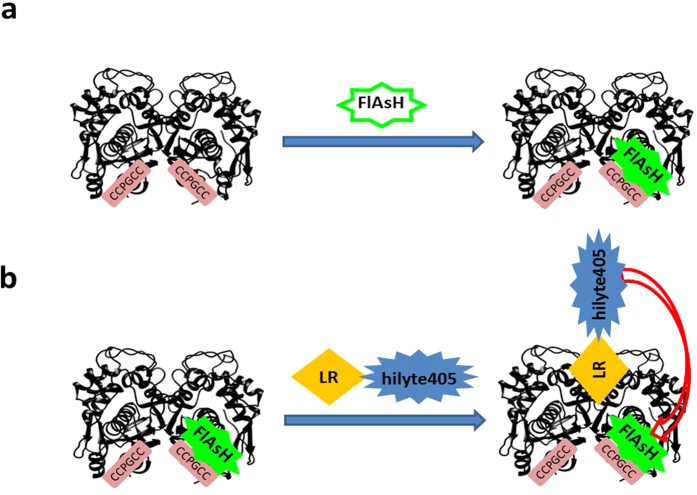
Overview of the FRET-based approach used to monitor hTS-LR peptide binding. (**a**) Schematic representation of the introduction of the TC motif (CCPGCC) near the N terminus of hTS and the subsequent binding of a green-emitting fluorescein-based diarsenical probe (FlAsH-EDT2) to form the FlAsH-TC-hTS complex. (**b**) Following hTS/LR recognition, excitation of LR-hilyte405 induces energy transfer to green-emitting FlAsH in the FlAsH-TC-hTS/LR-hilyte405 complex. This view represents a horizontal section of the dimer.
