# Supplementary material for: Intracellular quantitative detection of human thymidylate synthase engagement with an unconventional inhibitor using tetracysteine-diarsenical-probe technology
Source: Sci Rep. 2016 Jun 2;6:27198. doi: 10.1038/srep27198 (PMC4890114; doi:10.1038/srep27198)
Supplement: Supplementary Information [file srep27198-s1.pdf]

Intracellular quantitative detection of human thymidylate synthase engagement with an unconventional inhibitor using tetracysteine-diarsenical-probe technology

Glauco Ponterini[1,*], Andrea Martello[1,4], Giorgia Pavesi[1], Angela Lauriola[2], Rosaria Luciani[1], Matteo Santucci[1], Michela Pelà[3], Gaia Gozzi[2], Salvatore Pacifico[3], Remo Guerrini[3], Gaetano Marverti[2], Maria Paola Costi[1,*] and Domenico D'Arca[2,*].

[1] University of Modena and Reggio Emilia, Department of Life Sciences, Via Giuseppe Campi 183, Modena, 41125, Italy

[2] University of Modena and Reggio Emilia, Department of Biomedical, Metabolic and Neural Sciences, Via Giuseppe Campi 287, Modena, 41125, Italy

[3] University of Ferrara, Department of Chemical and Pharmaceutical Sciences, Via Fossato di Mortara 17-19, Ferrara, 44100, Italy

[4] University of Edinburgh, University/British Heart Foundation Centre for Cardiovascular Science, The Queen's Medical Research Institute, Edinburgh, EH16 4TJ, UK

Corresponding authors:

*Domenico D'Arca, domenico.darca@unimore.it;

*Maria Paola Costi, mariapaola.costi@unimore.it;

*Glauco Ponterini, glauco.ponterini@unimore.it

SUPPLEMENTARY RESULTS

SUPPLEMENTARY FIGURES

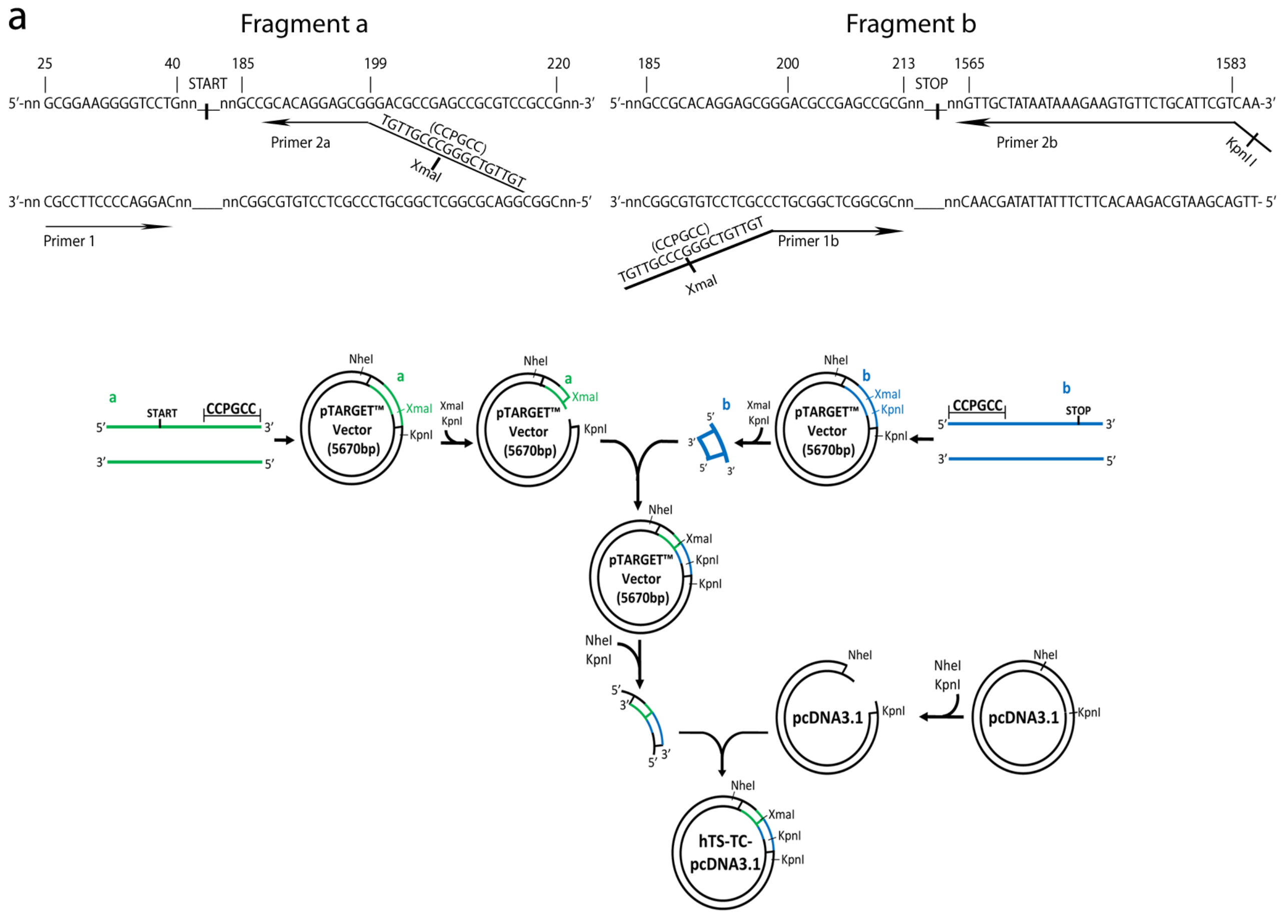

b

CCPGCC

```
MPVAGSELPR RPLPPAAQER DAEPRPPHGE LQYLGQIQHI LRCGVRKDDR TGTGTLSVFG
MQARYSLRDE FPLLTTKRVF WKGVLEELLW FIKGSTNAKE LSSKGVKIWD ANGSRDFLDS
LGFSTREEGD LGPVYGFQWR HFGAEYRDME SDYSGQGVDQ LQRVIDTIKT NPDDRRIIMC
AWNPRDLPLM ALPPCHALCQ FYVVNSELSC QLYQRSGDMG LGVPFNIASY ALLTYMIAHI
TGLKPGDFIH TLGDAHIYLN HIEPLKIQLQ REPRPFPKLR ILRKVEKIDD FKAEDFQIEG
YNPHPTIKME MAV
```

Supplementary Figure 1. Cloning strategy for the generation of the hTS-TC-pcDNA3.1 plasmid: (**a**) generation of the hTS-TC-pcDNA3.1 plasmid, containing TC-motif (CCPGCC), by insertion a and b fragments, into a pcDNA3.1plasmid; (**b**) Aminoacid sequence of hTS with TC-motif inserted between the amminoacids 20th and 21th.

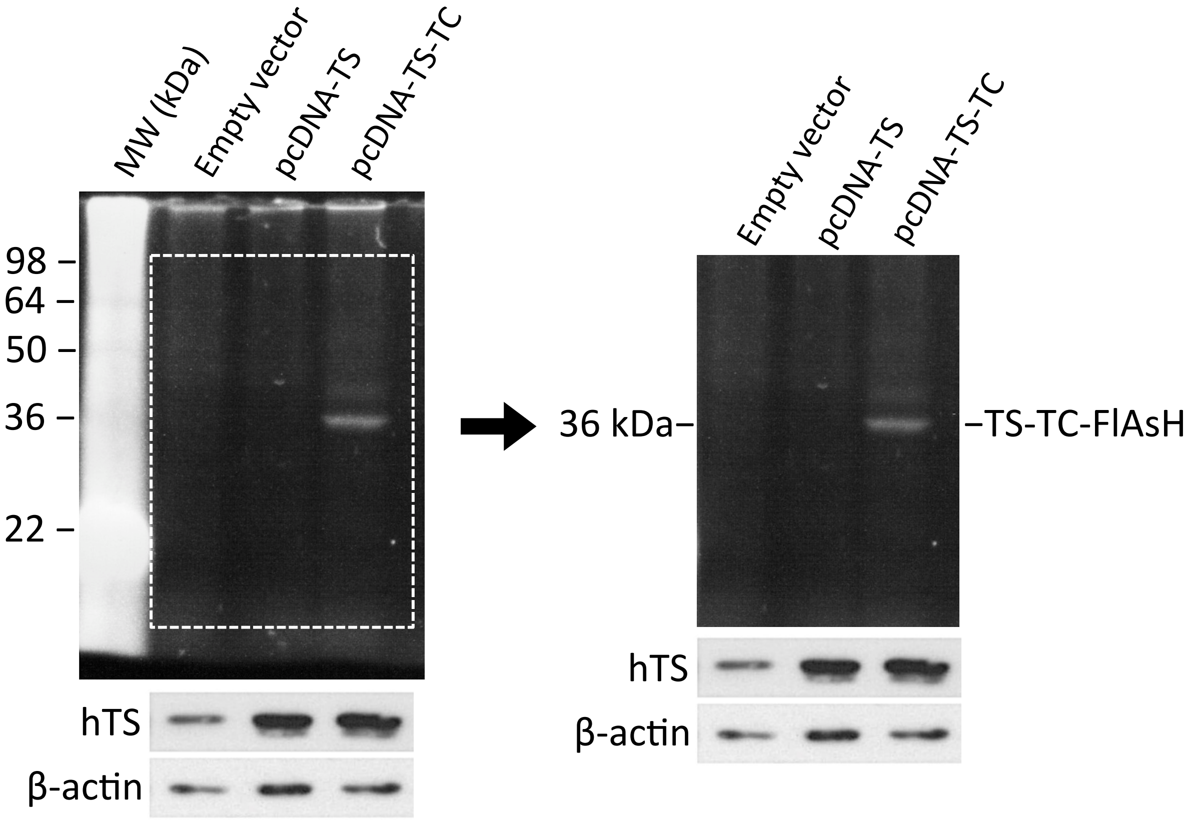

Supplementary Figure 2. Representative picture of polyacrylamide-gel for hTC-TC-FlAsH. (left) full-length polyacrylamide-gel detection for hTC-TC-FlAsH; (right) the same gel cropped as displayed as cropped gels in the main text of the manuscript in Figure 2b, the gel have been run by using different sample preparation: 10 mM TCEP (tris(2 carboxyethyl)phosphine) as the reductant instead of BME (β-mercaptoethanol) was added to the lysate before FlAsH-EDT2 labeling. In the bottom is shown the Western blot for hTS protein expression by using the same samples, but with BME as reductant.

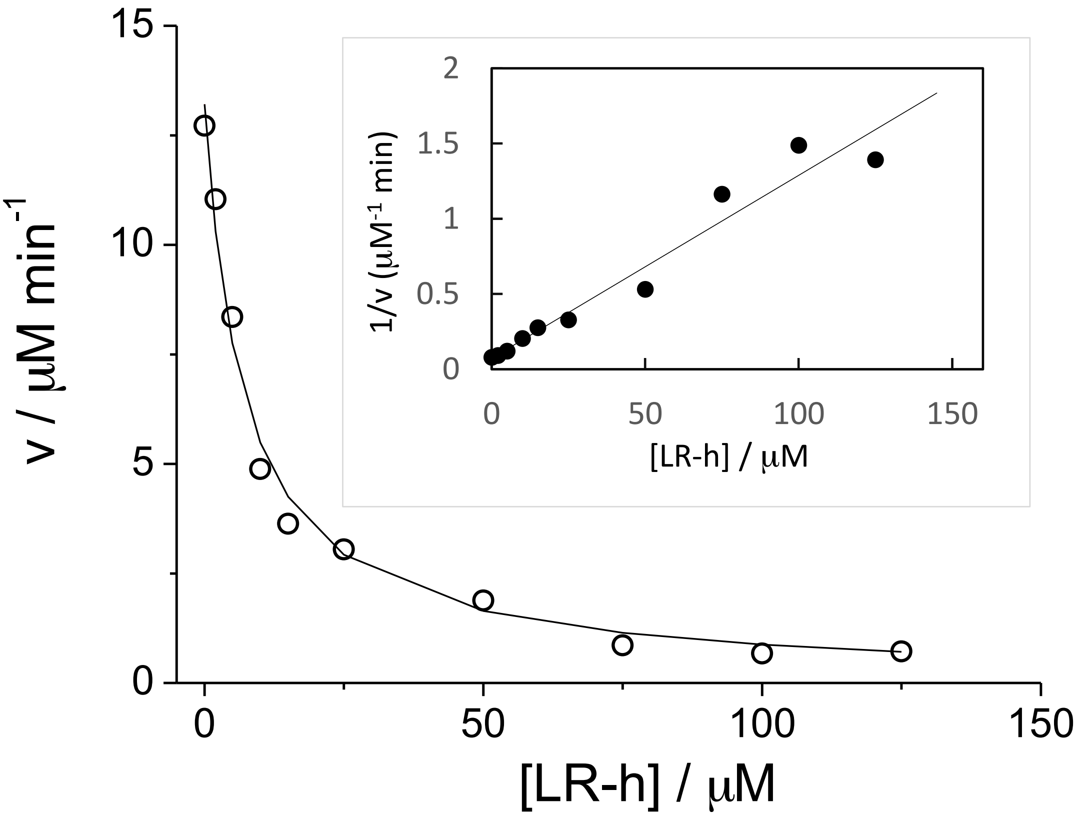

Supplementary Figure 3. Dependence of the activity of recombinant hTS on the concentration of LR-hilyte405. v = initial reaction rate. Inset: Dixon-type plot of the same data. The IC_{50} value (the [LR-h] at which the reaction rate was half that in the absence of inhibitor, 7±1 μM) was obtained from a linear least-squares fitting of the data in this plot.

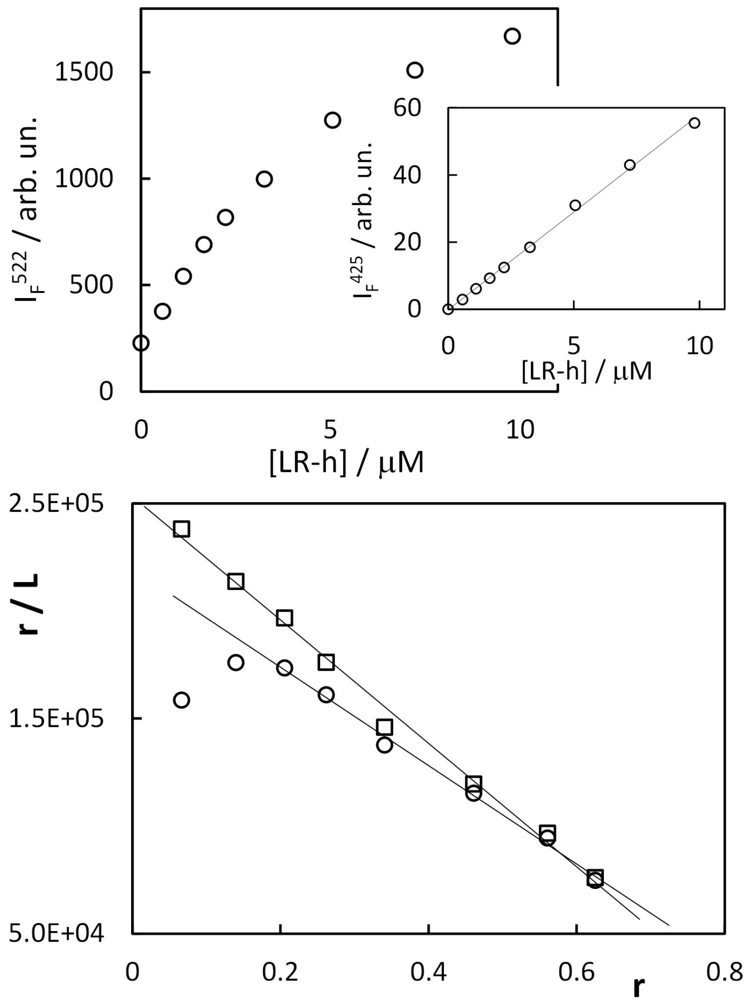

Supplementary Figure 4. FRET experiments on cell lysates with added hTS-F. Top: emission intensities of hilyte405 (inset, I_F^{425}), and fluorescein (I_F^{522}) as functions of the concentration of LR-h added to a HEK cell lysate to which hTS-F had been added at a concentration of 2.5 μM. Bottom: Scatchard plots of the raw data (circles) and of the data corrected for the presence of a high affinity binder at a concentration 140 nM (squares). r = fraction of occupied binding sites, L = molar concentration of free LR-h ligand .

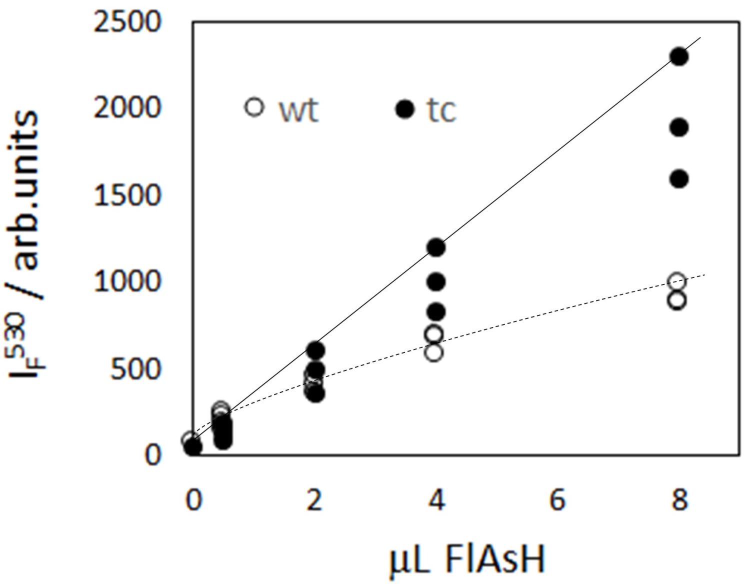

Supplementary Figure 5. Emission intensity from FlAsH after four 2 μl additions, each corresponding to 0.15 μM, to lysates of HEK cells, either wild-type (wt) or hTS-tetracys transfected (tc). Values immediately after addition and 10 and 40 minutes later are reported for each added amount. Values added to the tc cells increase with time.

SUPPLEMENTARY TABLE

Supplementary Table 1. Sequences of PCR primers for construction of the Tetracysteine-hTS tag and unmodified hTS plasmids.

Sequence	Oligonucleotides (5'-3')	Restriction site
Primer 1	5'-GCGGAAGGGGTCCTG-3'	
Primer 2b	5'-CTCGGTACCGACGAATGCAGAACACTTCTTTATTATAGC-3'	Kpn I
Primer 2a	5'- ACAACAGCCCGGGCAACACCGCTCCTGTGCGGC -3'	Xma I
Primer 1b	5'- TGTTGCCCGGGCTGTTGTGACGCCGAGCCGCG -3'	Xma I
